# Mutant Huntingtin affects toll-like receptor 4 intracellular trafficking and cytokine production in mast cells

**DOI:** 10.1186/s12974-020-01758-9

**Published:** 2020-03-27

**Authors:** Marian Jesabel Pérez-Rodríguez, Alfredo Ibarra-Sánchez, Abraham Román-Figueroa, Francisca Pérez-Severiano, Claudia González-Espinosa

**Affiliations:** 1 Departamento de Farmacobiología, Centro de Investigación y de Estudios Avanzados del IPN, Calzada de los Tenorios 235, Granjas Coapa, Tlalpan, 14330 Mexico City, Mexico; 2 Laboratorio de Neurofarmacología Molecular y Nanotecnología, Instituto Nacional de Neurología y Neurocirugía “Manuel Velasco Suárez”, Insurgentes Sur 3877, La Fama, Tlalpan, 14269 Mexico City, Mexico

**Keywords:** Huntingtin, Mast cells, TLR-4, Signaling, Intracellular receptor trafficking

## Abstract

**Background:**

Huntington’s disease (HD) is caused by the expression of a mutated variant of Huntingtin (mHtt), which results in the complex pathology characterized by a defective function of the nervous system and altered inflammatory responses. While the neuronal effects of mHtt expression have been extensively studied, its effects on the physiology of immune cells have not been fully described. Mast cells (MCs) are unique tissue-resident immune cells whose activation has been linked to protective responses against parasites and bacteria, but also to deleterious inflammatory allergic reactions and, recently, to neurodegenerative diseases.

**Methods:**

Bone marrow-derived mast cells (BMMCs) were obtained from wild-type (WT-) and mHtt-expressing (R6/1) mice to evaluate the main activation parameters triggered by the high-affinity IgE receptor (FcεRI) and the Toll-like receptor (TLR) 4. Degranulation was assessed by measuring the secretion of β-hexosaminidase, MAP kinase activation was detected by Western blot, and cytokine production was determined by RT-PCR and ELISA. TLR-4 receptor and Htt vesicular trafficking was analyzed by confocal microscopy. In vivo, MC-deficient mice (*c*-*Kit*^*Wsh/Wsh*^) were intraperitonally reconstituted with WT or R6/1 BMMCs and the TLR4-induced production of the tumor necrosis factor (TNF) was determined by ELISA. A survival curve of mice treated with a sub-lethal dose of bacterial lipopolysaccharide (LPS) was constructed.

**Results:**

R6/1 BMMCs showed normal β-hexosaminidase release levels in response to FcεRI, but lower cytokine production upon LPS stimulus. Impaired TLR4-induced TNF production was associated to the lack of intracellular dynamin-dependent TLR-4 receptor trafficking to perinuclear regions in BMMCs, a diminished ERK1/2 and ELK-1 phosphorylation, and a decrease in *c*-*fos* and TNF mRNA accumulation. R6/1 BMMCs also failed to produce TLR4-induced anti-inflammatory cytokines (like IL-10 and TGF-β). The detected defects were also observed in vivo, in a MCs-dependent model of endotoxemia. R6/1 and *c*-*Kit*^*Wsh/Wsh*^ mice reconstituted with R6/1 BMMCs showed a decreased TLR4-induced TNF production and lower survival rates to LPS challenge than WT mice.

**Conclusions:**

Our data show that mHtt expression causes an impaired production of pro- and anti-inflammatory mediators triggered by TLR-4 receptor in MCs in vitro and in vivo, which could contribute to the aberrant immunophenotype observed in HD.

## Background

Huntington’s disease (HD) is a dominant, deleterious neurological condition caused by the synthesis of a mutated variant of the protein Huntingtin (Htt) [1]. Mutant Htt (mHtt) is characterized by the presence of a N-terminal poly-Q string that causes the protein to misfold, giving new activities to the polypeptide and its aggregates. Since mHtt is expressed in cells from different lineages, HD has been recognized as a complex pathology, clinically defined by brain alterations and changes in immune responses like inflammation [2]. While the effects of mHtt on neurons have been the matter subject of intense research, the effects of mHtt expression on non-neural cells have not been fully studied.

Mast cells (MCs) are multifunctional innate immune cells, characterized by the presence of numerous electrodense granules in their cytoplasm. In the embryo, MCs are derived from the yolk sac [3], and in adults they are generated in the bone marrow and migrate to vascularized tissues as immature precursors, to complete their differentiation under the influence of locally produced mediators [4]. They are involved in both physiological and pathological immune responses, since upon a proper stimulus they release pre-stored and newly synthesized regulators of inflammation such as histamine, serotonin, proteases, lipid-derived compounds, cytokines, and chemokines. Under physiological conditions, MCs play an important role in tissue homeostasis, host defense, and tissue repair [5, 6]. They express high levels of the high-affinity IgE receptor (FcεRI), which binds the Fc portion of immunoglobulin E. Upon receptor crosslinking when IgE molecules form complexes with specific antigens (IgE/Ag), MCs release the content of their cytoplasmic granules (like the enzyme β-hexosaminidase) in a reaction known as anaphylactic degranulation. Then, they secrete various cytokines and chemokines that are responsible for the late-phase of allergic reactions [7]. Due to their capacity to produce pro-inflammatory mediators, MCs have been implicated not only in acute immune reactions but also in chronic deleterious conditions, like the neuroinflammation linked to neurodegenerative diseases [8, 9].

Studies on murine models have demonstrated that MCs participate in the host defense against invading microbes [10], since they express multiple receptors detecting pathogen-associated molecular patterns (PAMPs) and damage-associated molecular patterns (DAMPS). The Toll-like receptor 4 (TLR-4) is expressed in MCs and is activated by several DAMPs and gram-negative bacteria-derived lipopolysaccharide (LPS) [11]. In vivo, TLR-4 triggering in MCs leads to a rapid and sustained release of the tumor necrosis factor (TNF), which favors mouse survival after the intraperitoneal (i.p.) administration of high LPS doses [5, 12]. After recognizing its ligand, TLR-4 in MCs activates a signaling cascade that triggers the activation of the MAP kinases ERK 1/2, p38, and JNK, as well as the IκB kinase (IKK); this leads to the nuclear translocation of the transcription factors ELK-1 and p65 nuclear factor kappa-light-chain-enhancer of activated B cells (NFκB), respectively [13, 14]. Those events initiate a transcriptional program that activates immediate early genes (such as *c*-*fos*) and genes encoding for pro-inflammatory cytokines (like TNF and interleukin 6, IL-6). Anti-inflammatory cytokines like interleukin 10 (IL-10) and the transforming growth factor β (TGF-β) are also produced [15]. On the other hand, IKK and MAPKs phosphorylate key proteins of the cytokine secretion machinery, such as the membrane SNARE protein SNAP23 [12, 16] and the metalloprotease ADAM 17 [17]. Recent evidence indicates that the coupling of TLR-4 to intracellular signaling pathways requires the activity of the GTPase dynamin [18]. In distinct cells, dynamin activity controls endocytosis and intracellular vesicle trafficking because it catalyzes the final step on the budding of clatrhin-coated vesicles from the plasma membrane and other intracellular compartments. Its inhibition by compounds like dynasore not only affects membrane recycling processes but also the activation of intracellular signaling pathways and the synthesis of cytokines in response to physiologic stimuli [18, 19]. While the activation of both FcεRI and TLR-4 in MCs lead to cytokine secretion, the molecular mechanisms involved have been proven to be different, since LPS treatment of MCs fails to trigger anaphylactic degranulation [20]. Despite the current knowledge on the molecular events leading to cytokine production in MCs, several key players in the process remain to be identified.

Htt is a cytoplasmic protein that has been associated to various essential functions in mammalian cells; the expression levels of mHtt are known to impact several aspects of cell function. Htt interacts with proteins involved in gene expression, intracellular transport, intracellular signaling, and metabolism [21]. For instance, recent evidence suggests that Htt may function as a scaffolding protein in dynamin-dependent endocytosis of membrane receptors and vesicular trafficking [22–24]. Htt participates in the intracellular transport of a variety of organelles, including synaptic precursor vesicles [25], autophagosomes [26], endosomes and lysosomes [27], brain-derived neurotrophic factor (BDNF)-containing vesicles [28], amyloid precursor protein (APP)-positive vesicles [29], and GABA-receptor-containing vesicles [30]. Various murine models have been used to study the role of Htt in subcellular and physiological responses. In particular, the R6/1 mouse strain is widely utilized because it expresses the exon 1 of the human mHtt gene with approximately 116 CAG repeats. Those mice show a relatively rapid onset and progression of a phenotype that includes weight loss, motor abnormalities, neuropathological sequels, a shortened lifespan, and the presence of mHtt aggregates in striatal neurons and other cells [31].

The hypothesis that Htt participates in the processes of signal transduction and cytokine production in MCs is herein tested. To do so, distinctive activation parameters were measured in bone marrow-derived mast cells (BMMCs) from wild-type (WT) mice and the mHtt-expressing mouse strain R6/1. The effects of mHtt expression on MC-mediated innate immunity responses were also analyzed in vivo.

## Methods

### Mice and genotyping

mHtt-expressing mice of the strain B6CBA-Tg (HDexon1)61Gpb/1 (R6/1) and the corresponding control (Htt-expressing, WT mice) B6CBAF1/J were purchased from The Jackson Laboratory (Bar Harbor, ME, USA), with stock numbers 002809 and 100011. For experiments in vivo, MC-proficient C57BL/6 J and MC-deficient *c*-*Kit*^*Wsh/Wsh*^ (Wsh) mice were also acquired from The Jackson Laboratory, with stock numbers 000664 and 005051. When required, animals were kept in a 12-h dark/light cycle in a sterile environment at the Unit for the Production and Use of Laboratory Animals (UPEAL) in the Center for Research and Advanced Studies (Cinvestav). Each animal was genotyped with polymerase chain reaction (PCR) commercial kits, using genomic DNA and following the kit manufacturer’s directions.

### Reagents and antibodies

RPMI 1640 medium, monomeric IgE (clone SPE-7), and *Escherichia coli* (serotype 026:B6) LPS, NP-40, β-mercaptoethanol (2-ME), phosphate-buffered saline (PBS, Cat. No. P4417), and all reagents required for agarose and polyacrylamide gels were purchased from Sigma-Aldrich (St. Louis, MO, USA). BMMC complete media components (HEPES, non-essential amino acids, glutamine, penicillin/streptomycin mixture), and fetal bovine serum (FBS) were purchased from Gibco-Life Technologies (Gaithersburg, MD, USA). Murine interleukin 3 (IL-3) and stem cell factor (SCF) were purchased from PeproTech (Rocky Hill, NJ, USA). PVDF was purchased from Perkin Elmer (Boston, MA, USA). FR180204 and BAY117085 were purchased from Tocris Bioscience (Bristol, UK). Antibodies against p-ERK 1/2 (Cat. No. 9101, recognizing pTyr 202/pTyr 204), p-p65 NF-κB (Cat. No. 3033, recognizing pSer 536), and p-IKKα/β (Cat. No. 2697, recognizing Ser 176/180) were purchased from Cell Signaling Technology (Danvers, MA, USA). Antibodies against p-ELK-1 (Cat. No. ab34270, recognizing p-S383) were purchased from Abcam (Cambridge, UK). Antibodies against TLR-4 receptor used for confocal microscopy were purchased from BioLegend (San Diego, CA, USA) (Cat. No. SA1521, recognizing TLR4/MD2), and those used for flow cytometry (Cat. No. SAB1300056, recognizing N-terminal region of TLR-4 receptor) were purchased from Sigma-Aldrich. Antibodies against Htt (Cat. No. MAB5374, recognizing glutamine repeats) were purchased from Merck-Millipore (Burlington, MA, USA). Antibodies against FcεRI (Cat No. 17-5898-80, against the α subunit) were purchased from eBioscience (Thermo Fisher Scientific, San Diego, CA, USA). Antibodies against β-actin (Cat. No. SC-81178, recognizing the C-terminus) were purchased from Santa Cruz Biotechnology (Dallas, TX, USA). For Western blot, secondary antibodies (anti-mouse and anti-rabbit) were purchased from Jackson ImmunoResearch (West Grove, PA, USA). PE-conjugated antibodies for flow cytometry were acquired from Amersham Pharmacia Biotech (Little Chalfont, UK). Donkey anti-goat antibody coupled to Alexa Fluor 488, goat anti-rabbit-Alexa Fluor 568, and donkey anti-rat antibody coupled to Alexa Fluor 594 were purchased from Thermo Fisher.

### BMMC generation and sensitization with IgE

Bone marrow samples were obtained from tibias and femurs of WT or R6/1 mice. Bone marrow cells were placed in RPMI 1640 medium supplemented with IL-3 (20 ng/mL), SCF (10 ng/mL), 10% FBS, 100 IU/mL of penicillin, 100 mg/mL of streptomycin, 2-ME 50 μM, and nonessential amino acids 1 mM. The cells were cultured at 37 °C under a 5% CO_2_ atmosphere for 5–7 weeks, replacing the medium every 5–6 days. BMMC differentiation was assessed by detecting FcεRI expression on the plasma membrane by flow cytometry; only cultures showing more than 98% of FcεRI-positive cells were used. Before manipulation, the cells were sensitized for 24 h with 100 ng/mL of anti-DNP IgE (Clone SPE7).

### Viability test

The Muse™ Count Viability Kit (Millipore) was used to determine cell viability. Briefly, 10^6^ WT or R6/1 BMMCs were re-suspended for 15 min in a solution containing the reagent MHC100102 (included in the kit) 1:10 dilution. Dot plots indicating the percentage of live cells in each sample were generated with the Viability Intuitive Software, included in the analyzer.

### Flow cytometry

For each condition, 10^6^ WT or R6/1 BMMCs were incubated with IgE (100 ng/mL) for 24 h. Then, the cells were collected and re-suspended in universal blocking reagent (BioGenex, San Ramon, CA) for 5 min at 4 °C. The cells were washed twice with 1× PBS and incubated with anti-FcεRI (1:500 in blocking buffer containing 1× PBS, 5 g/L BSA, 0.5 g/L sodium azide), and anti-TLR-4 N-terminal Ab (1:100 in blocking buffer) for 30 min at 4 °C. The cells were washed and incubated with a PE-coupled anti-IgG Ab (1:200 in blocking buffer). After 30 min of incubation at 4 °C, the cells were washed again and re-suspended in 1% paraformaldehyde (PFA). An anti-isotype staining was performed at the same time. The samples were analyzed in a CytoFLEX LX (B-R-V-Y-N-I) flow cytometer (Beckman Coulter, Brea, CA, USA), using the software CytExpert for data acquisition; plots showing PE^**+**^ populations were generated with the software Kaluza Analysis. An Attune flow cytometer (Beckton Dickinson, Franklin Lakes, NJ, USA) was used in some experiments.

### Scanning electron microscopy

Two million sensitized WT or R6/1 BMMCs were centrifuged to a pellet and washed two times with PBS. The supernatant was discarded and the cells were re-suspended in the PBS residue. Then, the cells were let to stand at room temperature for 10 min in positively charged glass cover slips and fixed with 2.5% glutaraldehyde for 1 h. After three washes with PBS, the samples were post-fixed with 1% osmium tetroxide (OsO_4_) for 1 h. The fixed samples were washed three more times with PBS and dehydrated in graded ethanol dilutions: first, 50% ethanol for 10 min, followed by 60% ethanol for 10 min, and successively by 70%, 80%, 90%, and 100% ethanol for 10 min. Then, the cells were put in the critical drying point, coated by the gold-argon method, and scanned in a JSM-6510LV scanning electron microscope (JEOL, Tokyo, Japan). Images were taken at 25 kV and 7500× and 5000× enlargements were obtained.

### Transmission electron microscopy

WT and R6/1 BMMCs were observed by transmission electron microscopy (TEM) following a previously described protocol [32]. Briefly, 2 × 10^6^ sensitized BMMCs were centrifuged to a pellet and fixed in 2.5% glutaraldehyde, left to stand for 60 min at room temperature, post-fixed in 1% OsO_4_ for 60 min, dehydrated in ethanol, and embedded in Spurr resin (DER 332, Unione Chimica Europea, Milan, Italy). Ultrathin sections (70-μm width) were obtained in an ultramicrotome (Ultracut UCT, Leica, Wetzlar, Germany), mounted on etched nickel grids, contrasted with uranyl acetate and lead citrate, and rinsed with distilled water. The grids were analyzed in a JEM-1400 transmission electron microscope (JEOL). Micrographs were taken with a Morada camera (Olympus Soft Imaging Solutions, Muenster, Germany).

### RNA extraction and RT-PCR

Two million IgE-sensitized WT or R6/1 BMMCs were stimulated as required, collected, and lysed using TRI-reagent (Sigma-Aldrich) according to the manufacturer’s instructions, for total RNA extraction and RT-PCR. cDNA was obtained using the RevertAid First Strand cDNA Synthesis Kit (Thermo Fisher Scientific). The following primers were used: TLR-4, sense 5′-GCAATGTCTGGCAGGTGTA-3′ and antisense 5′-CAAGGGATAAGAACGCTGAGA-3′ [33]; MD-2, sense 5′-ATGTTGCCATTTATTCTCTTTTCGACG-3′ and antisense 5′-ATTGACATCACGGCGCTGAATGATG-3′ [34]; CD-14, sense 5′-CGTCTAGAAGAACACCATCGCTGTAAAG-3′ and antisense 5′-CGTCTAGAAGAACACCATCGCTGTAAAG-3′ [35]; TNF, sense 5′-TTCTGTCTACTGAACTTCGGGGTGATCGGTCC-3′ and antisense 5′-GTATGAGATAGCAAATCGGCTGACGGTGTGGG-3′ [36]; GAPDH, sense 5′-TGAAGGTCGGTGTGAACGGATTTGGC-3′ and antisense 5′-CATGTAGGCCATGAGGTCCACCAC-3′ [37]; *c*-*fos*, sense 5′-CGGGTTTCAACGCCGACTA-3′ and antisense 5′-TGGCACTAGAGACGGACAGAT-3′ [38]; IL-10, sense 5′-ATGCAGGACTTTAAGGGTTACTTGGGTT-3′ and antisense 5′-ATTTCGGAGAGAGGTACAAACGAGGTTT-3′; TGFβ, sense 5′-CGCAACAACGCCATCTATGAGAAA-3′ and antisense 5′-TTGCAGGAGCGCACAATCATGTTG-3′ [39]. PCR products were resolved on 2% agarose gels and stained with ethidium bromide. Developed gels were documented in a MiniBIS Pro system (DNR Bio-Imaging Systems, Neve Yamin, Israel) and analyzed with the software Gel Quant Express.

### Anaphylactic degranulation assay

The release of the pre-stored granular mediator (β-hexosaminidase) was used as a degranulation indicator in BMMCs. Two-million IgE-sensitized WT or R6/1 cells were placed in 1 mL of Tyrode’s/BSA buffer (20 mM HEPES pH 7.4, 135 mM NaCl, 5 mM KCl, 1.8 mM CaCl_2_, 1 mM MgCl_2_, 5.6 mM glucose, and 0.05% BSA) at 37 °C and added with 2,4-dinitrophenol conjugated to human serum albumin (DNP-HSA). After stimulation, the cells were centrifuged at 4 °C and the supernatants were incubated in citrate buffer with p-nitrophenyl-*N*-acetyl–β-d-glucosaminide for 60 min at 37 °C. The reaction was quenched in carbonate buffer and the optical density (OD) was determined at 405 nm. Specific release was calculated as the percentage of total β-hexosaminidase content in a 0.5% Triton X-100 cell lysate after subtracting baseline degranulation in non-stimulated cells [40].

### Determination of cytokine secretion by ELISA

Two million WT or R6/1 IgE-sensitized BMMCs were stimulated as described above and centrifuged. The concentration of secreted TNF was measured in about 200 μL of supernatant with a cytokine-specific ELISA kit (PeproTech, Cat. No. 900-K54), following the manufacturer’s instructions. A standard curve was included in each determination [41].

### Western blot

Two million WT or R6/1 IgE-sensitized BMMCs were re-suspended in 1 mL of supplemented RPMI and stimulated as required at 37 °C. Then, the cells were centrifuged and lysed in 100 μL of 2× Laemmli buffer supplemented with 4 mM sodium ortho-vanadate and 0.28 M 2-ME. Protein aliquots were resolved by SDS-PAGE and transferred to polyvinylidene difluoride (PVDF) membranes. The membranes were blocked with 4% skimmed milk and incubated overnight with the primary antibodies (1:10,000) in TBS-T buffer (25 mM Tris, 0.9% NaCl, 0.05% Tween-20). The primary antibodies were removed and the membranes were washed three times with TBS-T before incubation with the respective secondary antibody (1:15,000 or 1:20,000). The membranes were washed three times with TBS-T, and protein bands were detected with the chemiluminescent HRP substrate (Millipore). A densitometric analysis was performed with the software Molecular Imager Universal Hood II (Software Image Lab v.5.0, Bio-Rad, Hercules, CA, USA).

### Immunofluorescence and confocal microscopy

After pre-treatment and stimulation, BMMCs were re-suspended in 150 μL of 1× PBS. The cells were placed on a glass slide and left to stand for 10 min at room temperature. Then, the cells were fixed with 4% PFA for 15 min, blocked for 2 h in a solution containing 1% BSA, 5% inactivated donkey serum, and 0.1% Tween 20, and incubated overnight with primary anti-HTT and anti-TLR-4 antibodies (1:500). The primary antibodies were removed by washing with PBS for 1 min, ten times. Then, the slides were incubated for 2 h with a rat anti-goat IgG (Alexa 568) antibody (1:500) and a goat anti mouse IgG (Alexa 488) antibody (1:500). The slides were incubated for 5 min with a DAPI solution (1:500) to detect cell nuclei and mounted in PVA-DABCO. The samples were observed under a Zeiss LSM-800 with Airyscan confocal microscope (Carl Zeiss, Oberkochen, Germany), using the software Zen 2.3 SP1 Blue Edition for image acquisition and analysis.

### Reconstitution of *c*-*Kit*^*Wsh*/*Wsh*^ mice with MCs

Eight-week-old *c*-*Kit*^*Wsh/Wsh*^ mice were i.p. injected with 2 × 10^6^ BMMCs obtained from WT or R6/1 mice as described above [12]. Briefly, mature BMMCs were re-suspended in 200 μL of Tyrode’s buffer and injected into the peritoneal cavity of *c*-*Kit*^*Wsh*/*Wsh*^ mice. Further experiments on these mice, designated as Wsh Rec WT or Wsh Rec R6/1, respectively, were conducted 4 weeks after reconstitution. A successful reconstitution was defined as the restoration of the capacity of rapid (1 h after stimulus) TNF production after LPS administration (1 mg/kg) in the peritoneal cavity.

### Murine model of sub-lethal endotoxemia and peritoneal washes

Mice were administered (i.p.) with either sterile isotonic saline solution (SS, 0.9% NaCl) or LPS (1 mg/kg). One hour after challenge, the animals were sacrificed by CO_2_ inhalation, and peritoneal washes were performed by injecting 2 mL of SS into the peritoneal cavity. The injection was followed by a gentle massage, and the fluid was recovered with a syringe after exposing the peritoneum. Peritoneal washes were centrifuged 5 min at 3500×*g*, and cell-free supernatants were kept at − 80 °C until TNF quantification with a specific ELISA kit (PeproTech, 900-K54), using about 100 μL of peritoneal wash and following the manufacturer’s instructions.

### Murine model of lethal endotoxemia

To compare the mortality of mice expressing normal or mutant Htt after LPS challenge, WT (B6CBAF1/J), R6/1 (B6CBA-Tg mice), Wsh (*c*-*Kit*^*Wsh*/*Wsh*^), Wsh Rec WT (*c*-*Kit*^*Wsh*/*Wsh*^ reconstituted with BMMCs derived from WT mice), and Wsh Rec R6/1 (*c*-*Kit*^*Wsh*/*Wsh*^ reconstituted with BMMCs derived from R6/1 mice) were administered i.p. with either SS or LPS (80 mg/kg). After LPS administration, the animals were monitored for 40 h, and survival rates were recorded as previously described [42].

### Statistical analysis

The results are presented as the mean ± SD of at least three independent experiments using different cell cultures. For assays in vivo, at least 4–6 animals were used for each treatment. Data from different groups and time-course curves for two treatments were analyzed by one-way or two-way analysis of variance (ANOVA), respectively, followed by Tukey’s test. All analyses were performed with GraphPad Prism v.8 (GraphPad Software, San Diego, CA, USA).

## Results

### Characterization of WT and R6/1 BMMCs

To explore the effects of the expression of mHtt on MCs activation, BMMCs were obtained from B6CBAF1/J (WT) and transgenic B6CBA-Tg R6/1 (R6/1) mice. Although cell cultures from R6/1 mice generated about 20% less mature MCs than those from WT mice (Suppl. Fig. 1, panel a), no differences were found in mature cell viability after five weeks of culture (Suppl. Fig. 1, panel b).

**Fig. 1:**
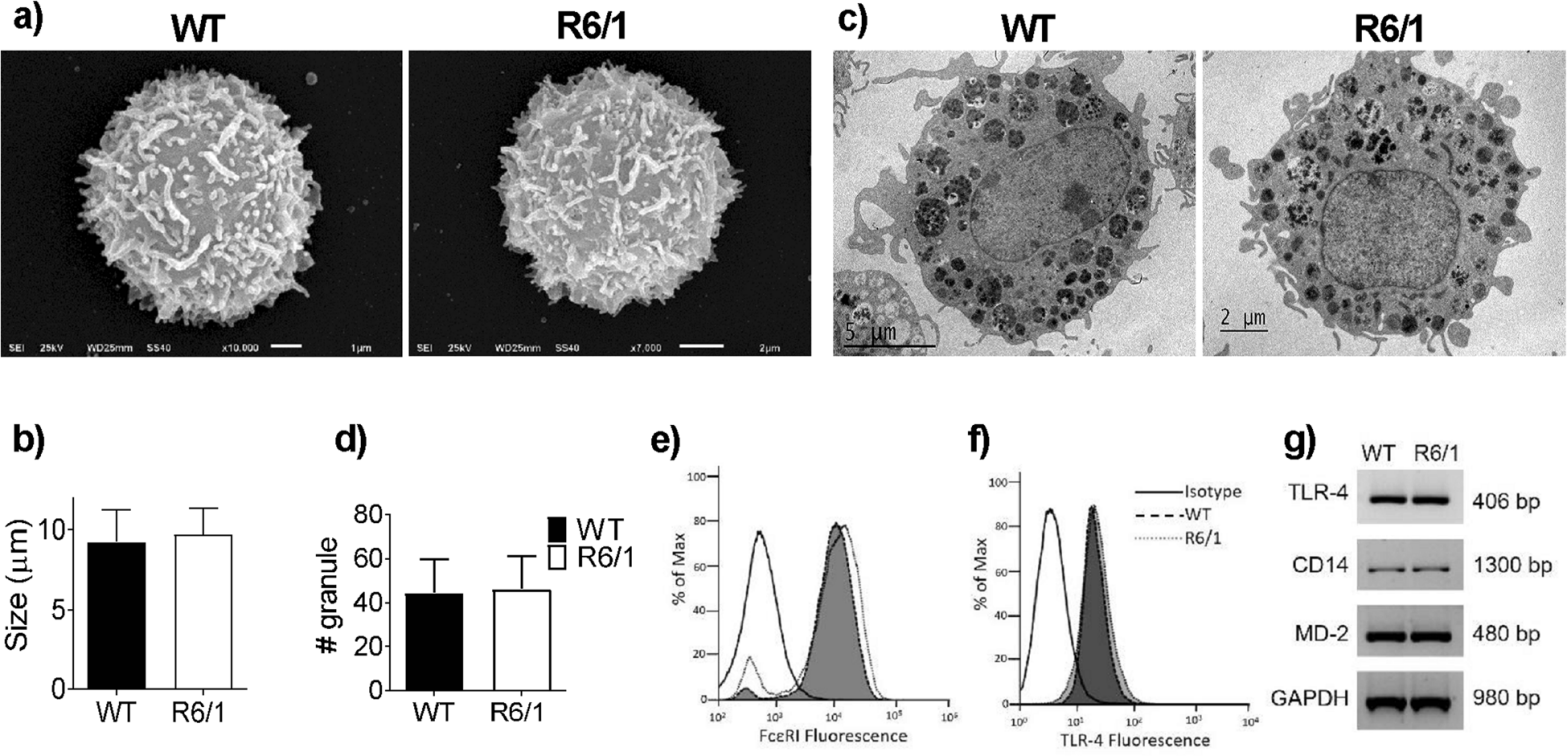
Characterization of WT and R6/1 BMMCs. Bone marrow-derived mast cells from WT or R6/1 mice were cultured as described in the Methods section. **a** Representative electronic scanning micrograph of WT and R6/1 BMMCs (10,000×). **b** Mean diameter of WT- and R6/1 BMMCs. **c** Representative electronic transmission micrograph of WT and R6/1 BMMCs. **d** Number of granules per cell in WT and R6/1 BMMCs. **e**, **f** Expression of FcεRI or TLR-4 receptors on the plasma membrane of WT and R6/1 BMMCs as determined by flow cytometry. **g** RT-PCR showing the expression of TLR-4, MD-2, and CD14 mRNAs in WT and R6/1 BMMCs. Analyses shown on (**b**) were performed by measuring 27–32 cells from at least three different cultures; analyses shown on (**d**) were performed by measuring 15–20 cells from at least three different cultures of each genotype by transmission electron microscopy. Data are presented as mean ± SD. Panels **e**, **f** show a representative flow cytometry plot from at least three experiments performed with different cell cultures. The image shown on panel **g** is representative of two experiments performed with different cell cultures

The morphology of BMMCs derived from WT and R6/1 mice was analyzed with various tools. As shown in Fig. 1a, b, electronic scan microscopy revealed that mature cells from both mouse strains were similar in mean size (9.25 ± 1.99 μm for WT cells and 9.71 ± 1.61 μm for R6/1 cells). Transmission electron microscopy showed the presence of numerous mature electrodense granules in WT and R6/1 BMMCs; no differences were found in the number of granules between genotypes (Fig. 1c, d). Since FcεRI receptor expression is a proven marker of the homogeneity and maturity of a BMMC culture [43], we analyzed the expression of this receptor in BMMCs from both mouse strains by flow cytometry. As shown in Fig. 1e, no differences were observed in the expression of this receptor on the membrane of both types of cells, and over 99% of cells in both cultures were FcεRI-positive. Also, when the expression of TLR-4 was assessed (Fig. 1f), the reported pattern of expression [44] was detected, and no significant differences were observed in mRNA expression for TLR-4, CD14, and MD-2 among the cell types (Fig. 1g).

### β-hexosaminidase release was unaffected after FcεRI crosslinking, but TNF production decreased after TLR-4 activation in R6/1 BMMCs

**Fig. 2:**
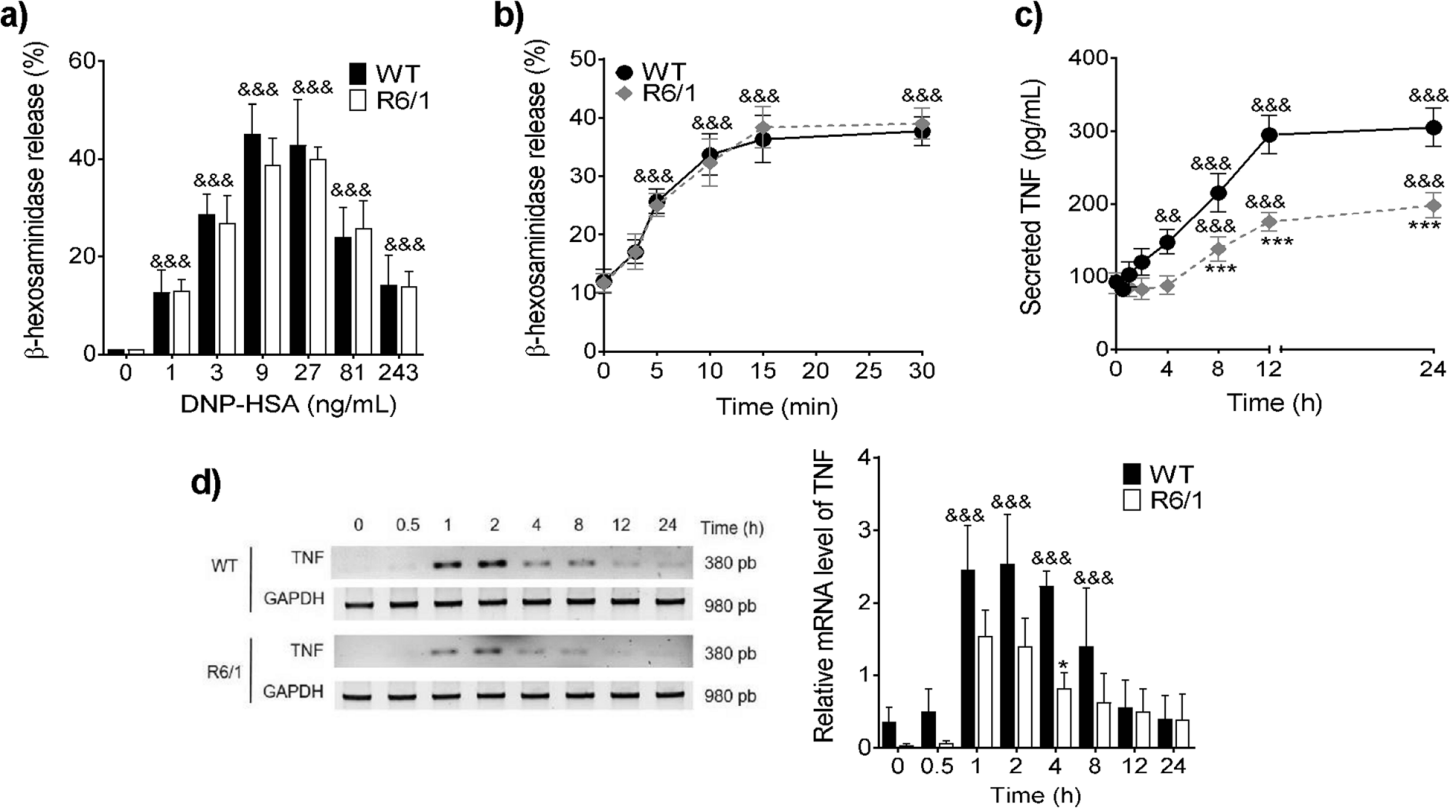
FcεRI-induced β-hexosaminidase release and TNF production after TLR-4 receptor activation in R6/1 BMMCs. **a** Dose-response curve of β-hexosaminidase release by BMMCs. **b** Time-course of β-hexosaminidase secretion by BMMCs after DNP-HSA (27 ng/mL) treatment. **c** Time-course of TNF secretion after LPS (500 ng/mL) treatment to WT and R6/1 BMMCs. TNF in cell supernatants was quantified by ELISA. **d** Time-course of TNF mRNA expression in WT and R6/1 cells after LPS treatment (500 ng/mL). Left panel shows a representative experiment; right panel shows densitometric analysis of different experiments. Data are presented as the mean ± SD of at least three independent experiments performed with different cell cultures. *p < 0.05, *** *p* < 0.001 vs. WT cells; ^&&^*p* < 0.01, ^&&&^*p* < 0.001 vs. the value at time zero or in non-stimulated cells, as determined by two-way ANOVA followed by Tukey’s test

Then, the possible differences in FcεRI-induced anaphylactic degranulation between both cell types were determined by measuring the secretion of pre-formed β-hexosaminidase when BMMCs were stimulated by IgE/Ag complexes. After sensitization with monoclonal anti-DNP IgE, the cells were incubated with the hapten dinitrophenol conjugated to human serum albumin (DNP-HSA) to induce FcεRI receptor crosslinking. As shown in Fig. 2a, BMMCs from WT and R6/1 mice secreted about 40% (43 ± 9.21% in WT cells and 40 ± 2.34% in R6/1 cells) of their β-hexosaminidase content in response to 27 ng/mL of antigen, with no significant differences between genotypes. In a time-course experiment, both cell types secreted a maximum amount of β-hexosaminidase after 30 min in the presence of 27 ng/mL of antigen (38 ± 2.51% in WT cells and 39 ± 2.64% in R6/1 cells, Fig. 2b).

Then, the responses triggered by TLR-4 stimulation in WT and R6/1 BMMCs were studied. Since β-hexosaminidase is not released by stimulating this receptor [45], TNF production was quantified instead. BMMCs were stimulated with 500 ng/mL of *E*. *coli* LPS, and TNF secretion was measured by ELISA in a time-course experiment. As shown in Fig. 2c, a significant TNF secretion was observed in WT BMMCs 4 h after LPS stimulus, and TNF concentration in supernatants reached a maximum value (295 ± 26.45 pg/mL) 12 h after stimulus. In contrast, LPS-induced TNF secretion in R6/1 BMMCs was only significant 8 h post-challenge and was lower than in WT BMMCs (max = 197.5 ± 17.07 pg/mL) at 24 h.

To determine whether TNF synthesis could be affected in R6/1 BMMCs, the accumulation of TNF mRNA in response to LPS was measured. As shown in Fig. 2d, an increase in TNF mRNA was observed as early as 1 h after LPS stimulus in WT BMMCs (a 2.4 ± 0.60-fold increase 1 h after); this increase was sustained 2 and 4 h after stimulus. In contrast, a lower increase in the levels of this mRNA was observed in R6/1 cells (a 1.53 ± 0.35-fold increase 1 h after and a 0.81 ± 0.21-fold increase 4 h after).

### Impaired TLR4-dependent activation of IKK, p-65, ERK1/2, and ELK-1, as well as *c*-*fos* mRNA accumulation in R6/1 BMMCs

The role of Htt in signaling pathways leading to TLR4-induced TNF mRNA accumulation and TNF secretion in BMMCs was also analyzed. Previously, we had reported that TNF secretion by BMMCs after LPS stimulus requires IKK to phosphorylate the SNARE protein SNAP-23 (necessary for the fusion of TNF-containing vesicles with the plasma membrane [44]) and MAPK ERK 1/2 to activate ADAM17 (the metalloproteinase that processes TNF at the plasma membrane [44, 46]); therefore, we analyzed the participation of IKK and ERK 1/2 in the accumulation of TNF mRNA in BMMCs. In the canonical TLR-4 signaling cascade described for dendritic cells (DCs) and macrophages, the activation of IKK and ERK1/2 controls the function of the transcription factors NFκB and ELK-1, respectively [47, 48], while NFκB has been associated with the transcription of the TNF gene. To test whether this could be the case for MCs, WT BMMCs were pre-treated with vehicle, the IKK inhibitor BAY117085, or the ERK1/2 inhibitor FR180204 before LPS stimulus, and a time-course experiment of TNF mRNA accumulation was conducted. As shown in Suppl. Fig. 2a, pre-incubation with BAY117085 failed to modify TLR4-triggered TNF mRNA accumulation, whereas FR180204 blocked the synthesis of TNF mRNA after LPS stimulus (Fig. S2, panel b). These results indicate that TNF mRNA accumulation after TLR-4 activation in BMMCs depends on the activity of the MAPK ERK 1/2, and not significantly on the function of IKK.

**Fig. 3:**
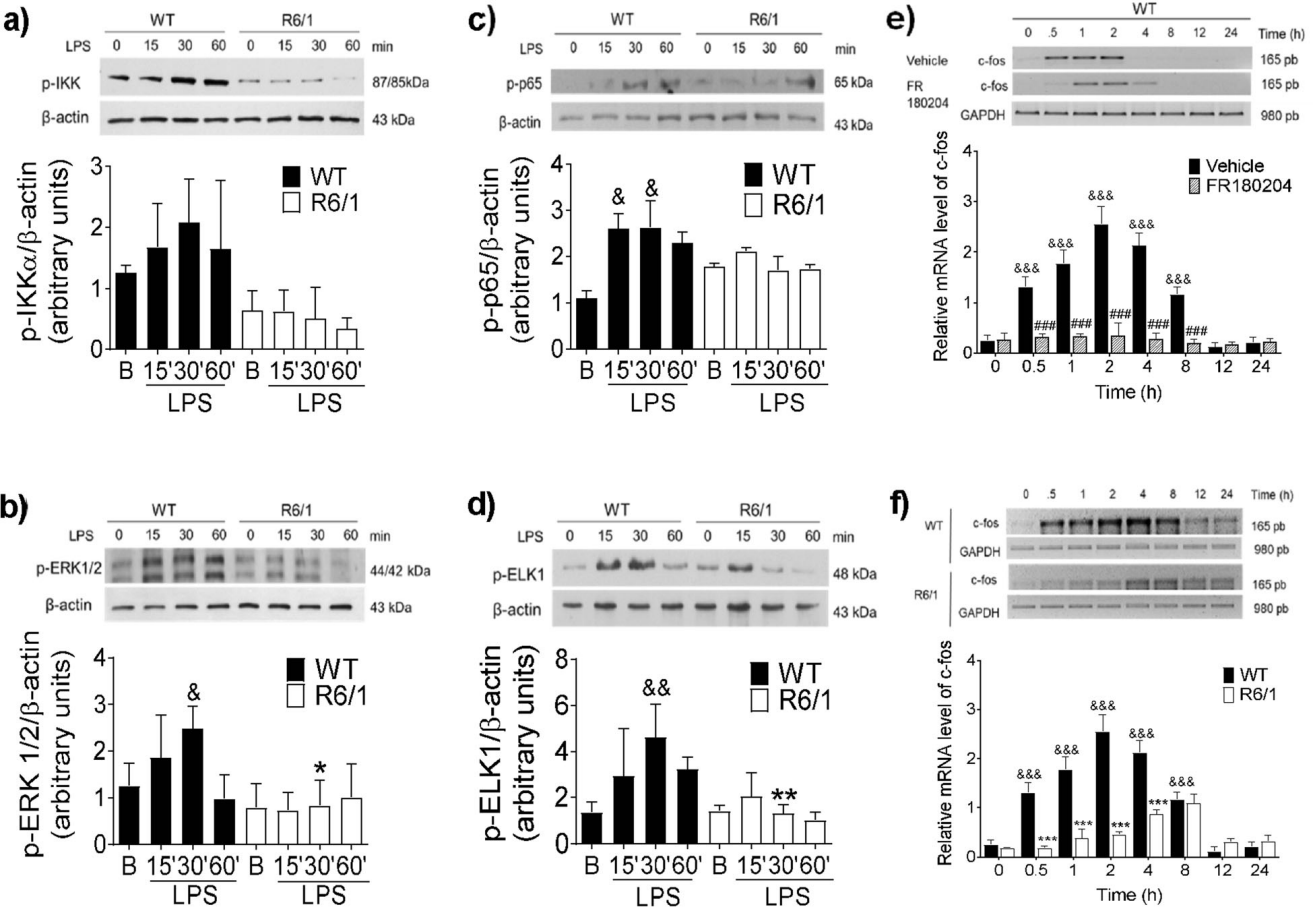
Impaired TLR-4 signaling in R6/1 BMMCs. WT and R6/1 BMMCs were treated with LPS (500 ng/mL), at 0, 15, 30, and 60 min after stimulus, the cells were isolated, and the phosphorylation of specific proteins was determined by Western blot. **a** Time-course of IKK, **b** ERK1/2, **c** p65, and **d** ELK-1 activation. **e** Time-course of *c*-*fos* mRNA accumulation in FR180204-treated WT BMMCs. Two-million WT BMMCs were pre-incubated for 15 min with FR180204 (10 μM) and stimulated with LPS (500 ng/mL). Total RNA was isolated at the indicated times and RT-PCR was performed. **f** Time-course of LPS-induced *c*-*fos* mRNA accumulation in WT and R6/1 BMMCs. The cells were treated with 500 ng/mL of LPS and total RNA was isolated at different times to perform RT-PCR. Data shown in the upper part of all panels are from a representative experiment, whereas the lower part shows the results of at least three independent experiments performed with different cultures. Data are presented as the mean ± SD, *p < 0.05, **p < 0.01, ***p < 0.001 vs. WT values; ^&^*p* < 0.05, ^&&^*p* < 0.01, ^&&&^*p* < 0.001 vs. to the value at time zero or in non-stimulated cells; #*p* < 0.05, ##*p* < 0.01, ###*p* < 0.001 vs. vehicle-treated cells, as determined by two-way ANOVA followed by Tukey’s test

Then, we analyzed the phosphorylation of IKKα/β, the p65 subunit of NFκB, and ERK1/2, as well as the ensuing phosphorylation of ELK-1, in LPS-stimulated WT and R6/1 BMMCs. As shown in Fig. 3a, LPS treatment led to IKK phosphorylation in WT BMMCs 30 min after the stimulus (a 1.6-fold increase). In contrast, no significant phosphorylation was observed in cells from R6/1 mice (0.03-fold increase) at that time. A similar decrease was observed in ERK1/2 phosphorylation. WT BMMCs showed activation of that kinase 30 min after stimulus (1.79-fold increase), whereas this effect was not observed in R6/1 cells (0.27-fold increase) at that time (Fig. 3b). On the other hand, the phosphorylation of the p65 NFκB subunit was observed in WT cells, reaching a maximum value 30 min after stimulus (2.62-fold increase). In contrast, significant phosphorylation was observed in R6/1 cells in basal conditions (1.78-fold increase with respect to the basal value in WT cells), and did not increase after LPS stimulus (Fig. 3c). Furthermore, ELK-1 phosphorylation was observed in response to LPS, reaching a maximum value 30 min after the stimulus in WT cells (4.62-fold increase) but not in R6/1 BMMCs (Fig. 3d). A quantification of total amounts of ERK1/2, IKK, ELK-1, and p65 by Western blot revealed no differences in the quantities of those proteins between both genotypes (data not shown).

To confirm the impaired ERK1/2 activation observed in R6/1 BMMCs, the functional response dependent on ELK-1 activation was assessed by measuring the production of *c*-*fos* mRNA (an immediate early gene whose synthesis depends on that transcription factor [49]). First, we confirmed that the synthesis of *c*-*fos* mRNA depends on the activation of ERK 1/2 in WT BMMCs by evaluating the effect of the ERK 1/2 inhibitor FR180204 on LPS-induced *c*-*fos* mRNA accumulation. As shown in Fig. 3e, pre-treatment of cells with FR180204 delayed the accumulation and reduced the maximum levels of *c*-*fos* mRNA synthesis in LPS-stimulated WT cells. On the other hand, as expected from an impaired ERK 1/2 phosphorylation in R6/1 BMMCs, *c*-*fos* mRNA accumulated in WT after LPS stimulus, while this accumulation was delayed in mHtt-expressing BMMCs and it failed to reach its maximum value (Fig. 3f). Finally, to corroborate the decrease in the activation of IKK- and ERK1/2-dependent pathways induced by mHtt expression in BMMCs, we analyzed the TLR4-dependent synthesis of IL-10 and TGF-β, two anti-inflammatory cytokines whose synthesis relies on the activation of the transcription factors AP-1 and NFκB in immune cells [50]. As shown in Suppl. Fig. 3, a rapid accumulation of IL-10 and TGF-β mRNAs was observed in WT BMMCs upon LPS stimulus. However, none of those mRNAs was detected at the tested times when R6/1 BMMCs were treated with LPS.

### Dynamin activity mediates TLR4-dependent ERK 1/2 phosphorylation, *c*-*fos* and TNF mRNA accumulation, and TNF secretion

Htt has been reported to participate in the signal transduction systems of several receptors [51]. Its role is mainly related to the process of vesicular trafficking and receptor internalization required in certain cell types for kinase and transcription factor activation. For example, in striatal neurons, Htt contributes to the retrograde transport of the BDNF receptor TrkB, leading to ERK 1/2 activation and *c*-*fos* transcription [27]. After observing a defective ERK1/2 signaling cascade in R6/1 BMMCs, we decided to investigate whether TLR-4 vesicular trafficking was necessary for ERK 1/2 activation and TNF mRNA production in WT cells, and whether these events could be affected in mHtt-expressing BMMCs.

**Fig. 4:**
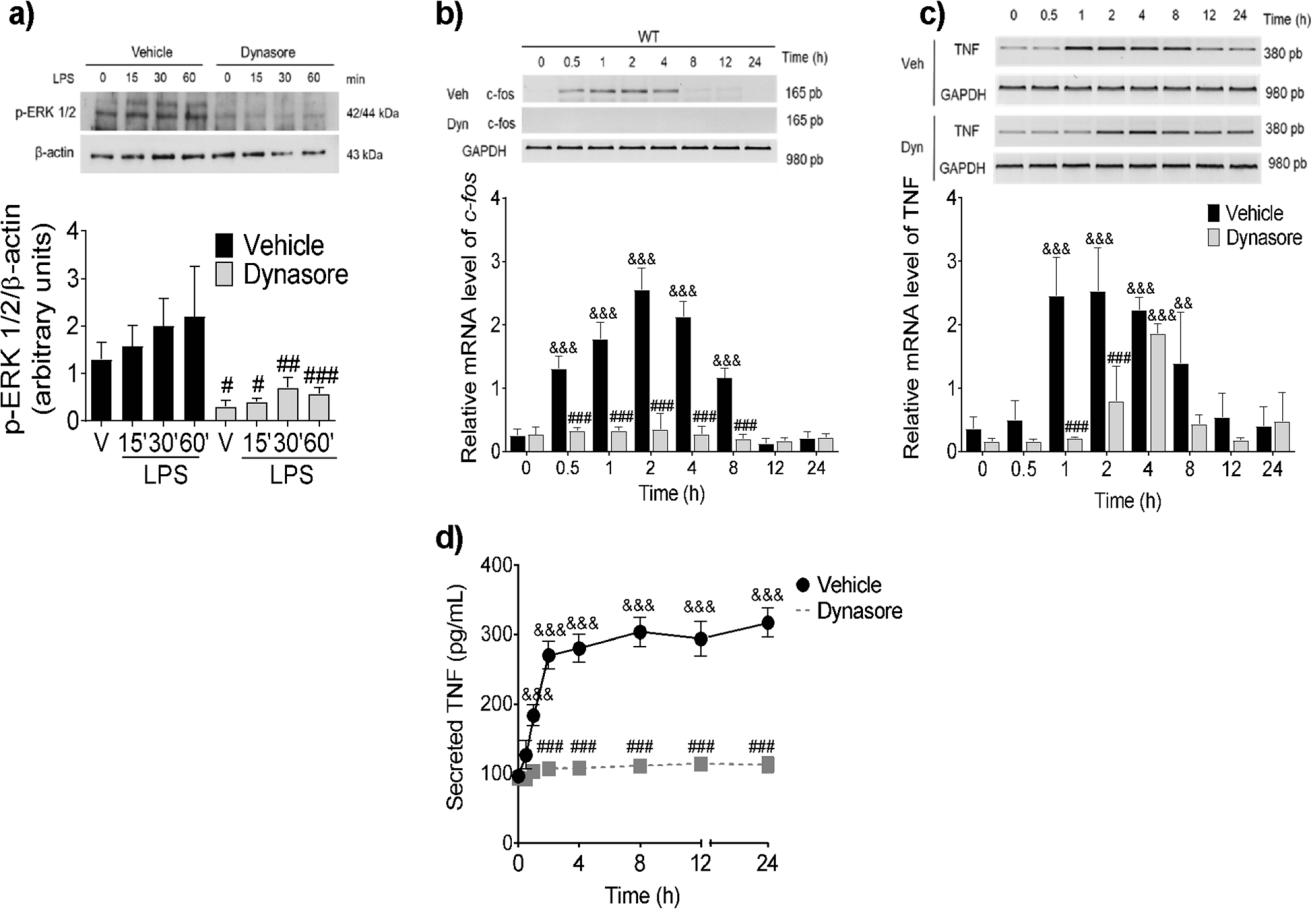
Dynamin activity mediates TLR4-dependent ERK 1/2 phosphorylation, *c*-*fos* and TNF mRNA accumulation and TNF secretion. WT BMMCs were pre-incubated with dynasore (80 μM) for 30 min before LPS addition (500 ng/mL). After stimulus, the cells were incubated at 37 °C for the indicated times and processed to obtain total protein or total RNA for RT-PCR. **a** ERK1/2 phosphorylation, **b***c*-*fos* mRNA accumulation, **c** TNF mRNA synthesis, and **d** TNF secretion. Data are presented as the mean ± SD of 3–4 independent experiments. ^&&^*p* < 0.01, ^&&&^*p* < 0.001 vs. the value at time zero or in non-stimulated cells; #*p* < 0.05, ##*p* < 0.01, ###*p* < 0.001 vs. vehicle-treated cells, as determined by two-way ANOVA and post-hoc Tukey’s test

We tested the effect of dynasore, an inhibitor of the dynamin- and clathrin-dependent internalization [52], on the TLR4-induced activation of ERK 1/2 in WT BMMCs. As shown in Fig. 4a, while LPS induced ERK 1/2 phosphorylation in vehicle-treated cells (2.2-fold increase), such effect was not observed when the cells were pre-incubated with dynasore. Dynasore pre-treatment also prevented the LPS-dependent accumulation of *c*-*fos* and TNF mRNAs to reach its maximum value (Fig. 4b, c) and the subsequent TNF secretion in WT BMMCs (Fig. 4d).

### LPS treatment triggers dynamin-dependent vesicular trafficking of TLR-4 receptor in WT BMMCs

**Fig. 5:**
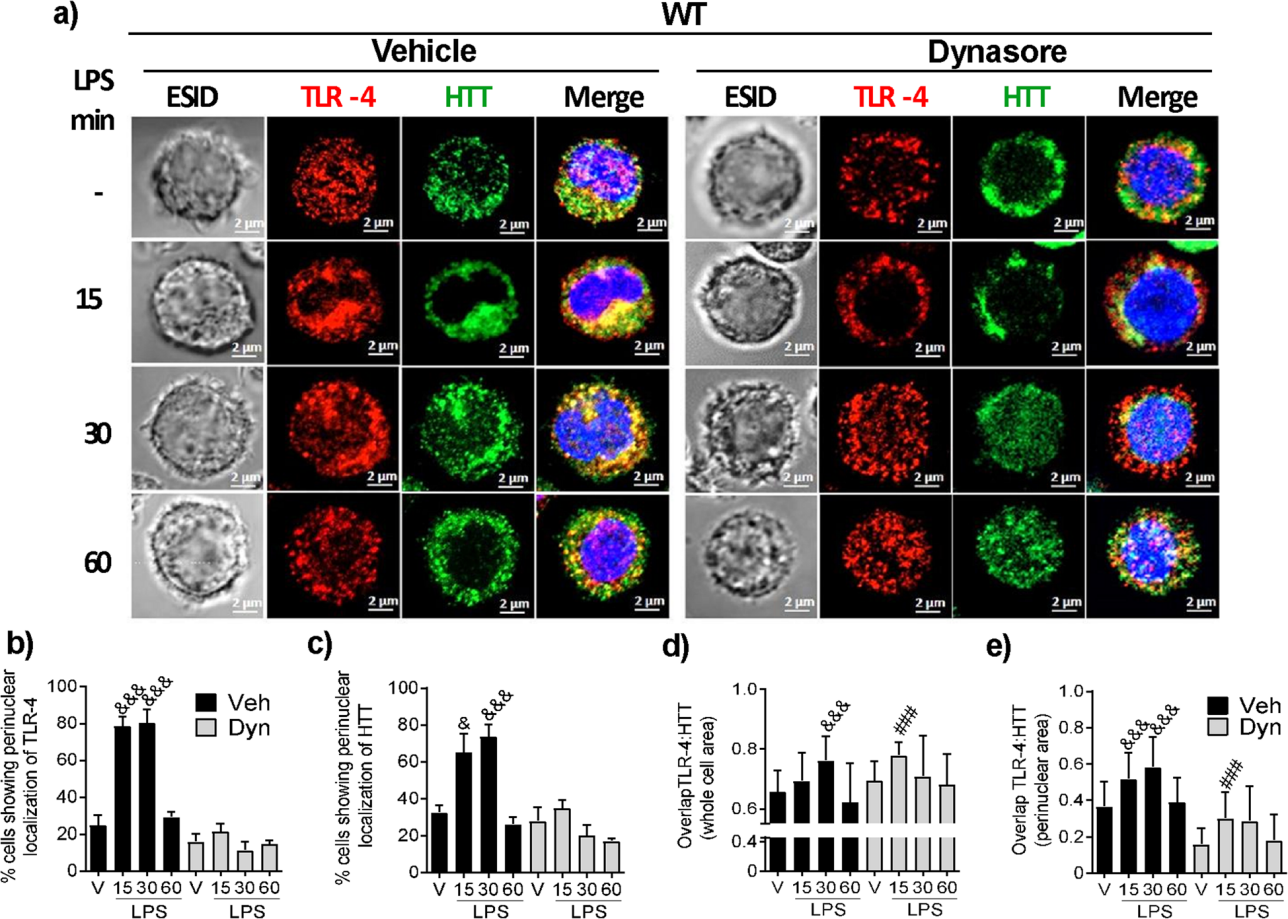
LPS treatment causes dynamin-dependent TLR-4 vesicular trafficking in WT BMMCs. The cells were pre-incubated for 30 min with either vehicle or dynasore (80 μM) and then stimulated with LPS (500 ng/mL). The cells were fixed and processed for confocal microscopy 15, 30, or 60 min after stimulus. **a** Representative photographs of cells showing TLR-4 (Red), Htt (green), and nuclei (blue). For reference, an ESID photograph is shown in each time point. Representative images are shown (*n* = 60–70 cells analyzed per condition). The images were taken at × 63 magnification. Scale bar = 2 μm. **b**, **c** Percentage of cells exhibiting perinuclear localization of TLR-4 and Htt, respectively. Pearson’s correlation coefficient for TLR-4 and Htt in **d** the whole area and in **e** the perinuclear area of the images obtained from the equatorial slices. Data are presented as the mean ± SD of at least three independent experiments, where 60–70 individual cells in three fields per preparation were analyzed. ^&^*p* < 0.05, ^&&&^*p* < 0.001 vs. the value at time zero or in non-stimulated cells; ###*p* < 0.001 vs. vehicle-treated cells as determined by two-way ANOVA and post hoc Tukey’s test

To further analyze the possible internalization and vesicular trafficking of TLR-4 receptor in BMMCs and a potential participation of Htt in the phenomenon, the cellular localization of both molecules was analyzed in WT BMMCs by confocal microscopy. As shown in Fig. 5a, under basal conditions, while TLR-4 was barely found on the cell membrane of WT BMMCs, it was often detected in intracellular locations. However, 15 and 30 min after LPS treatment, a significant fraction of TLR-4 molecules was relocated to the perinuclear cloud in about 80% of the cells (Fig. 5b). On the other hand, in vehicle-treated cells, Htt was detected in nuclear and cytoplasmic locations, in a pattern resembling a microtubule network and associated to intracellular vesicles. After LPS stimulus, Htt was redistributed to the perinuclear cloud in about 80% of analyzed cells (Fig. 5a, c).

In contrast, when the cells were pre-treated with dynasore, TLR-4 receptor was found in larger aggregates distributed throughout the cytoplasm of vehicle-treated cells. Furthermore, no significant translocation to perinuclear areas was observed upon LPS stimulus (Fig. 5a, right panel). After dynasore treatment, Htt was found in aggregates near to the plasma membrane and, upon LPS stimulus, its translocation to perinuclear areas was decreased in most cells (Fig. 5c).

Regarding to the association of TLR-4 and Htt in WT cells, an increased co-localization of the receptor with Htt (as determined by Pearson’s coefficient) was observed in the whole cell area after LPS stimulus; this co-localization was not significantly affected by dynasore treatment (Fig. 5d). However, the extensive co-localization of TLR-4 and Htt in the perinuclear region after LPS treatment was significantly decreased by dynasore (Fig. 5e).

### Impaired LPS-induced TLR-4 and Htt vesicular trafficking in R6/1 BMMCs

**Fig. 6:**
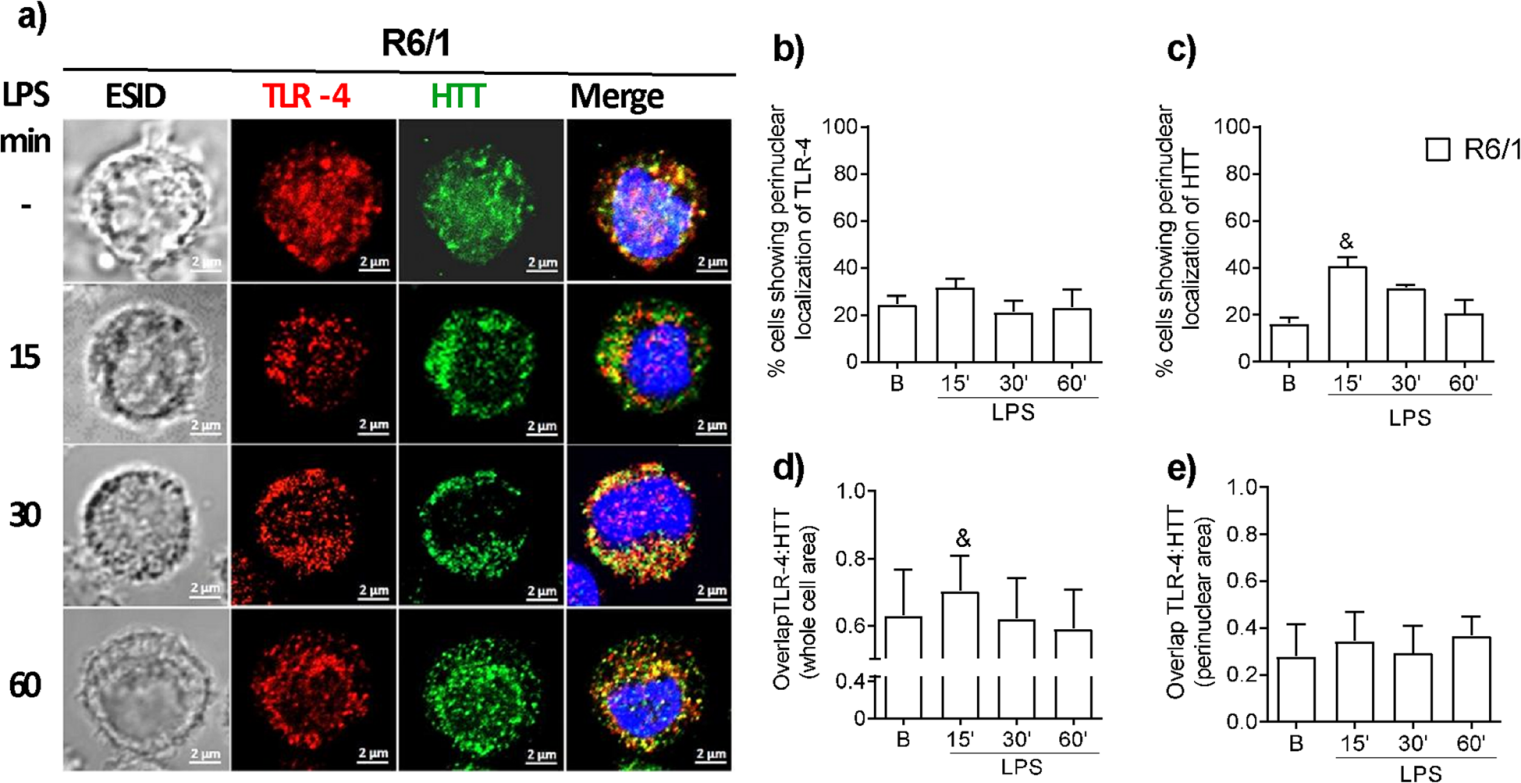
Impaired LPS-induced TLR-4 and Htt vesicular trafficking in R6/1 BMMCs. The cells were stimulated with LPS (500 ng/mL) and then processed for confocal microscopy to detect TLR-4 and Htt at 0, 15, 30, and 60 min after stimulus. **a** Representative images (*n* = 60–70 cells per condition) are shown. The images were taken at a × 63 magnification. Scale bar = 2 μm. TLR-4 is shown in red, Htt is shown in green, and cell nuclei are shown in blue. **b** Percentage of cells with a perinuclear localization of TLR-4. **c** Percentage of cells showing a perinuclear localization of Htt. Pearson’s correlation coefficient for TLR-4 and Htt in **d** the whole area and in **e** the perinuclear area of the images obtained from the equatorial slices. Data are presented as the mean ± SD (*n* = 4). ^&^*p* < 0.05 vs. the value at time zero or in non-stimulated cells as determined by two-way ANOVA and post hoc Tukey’s test

The hypothesis that TLR-4 receptor internalization could be affected by mHtt was tested by assessing the co-localization of Htt and TLR-4 after LPS stimulus by confocal microscopy in R6/1 BMMCs. As shown in Fig. 6a, TLR-4 was located in intracellular compartments in R6/1 cells, just like in WT BMMCs. However, the LPS-induced translocation of TLR-4 to perinuclear compartments was significantly decreased in comparison to WT BMMCs (Fig. 6b). On the other hand, an increase in the perinuclear translocation of Htt was observed in R6/1 cells after LPS stimulus (Fig. 6c). As expected, no significant co-localization of TLR-4 with Htt in the cytoplasm or perinuclear areas of R6/1 BMMCs was found (Fig. 6d, e).

### In vivo effects of impaired TLR-4 signaling in R6/1 mast cells

**Fig. 7:**
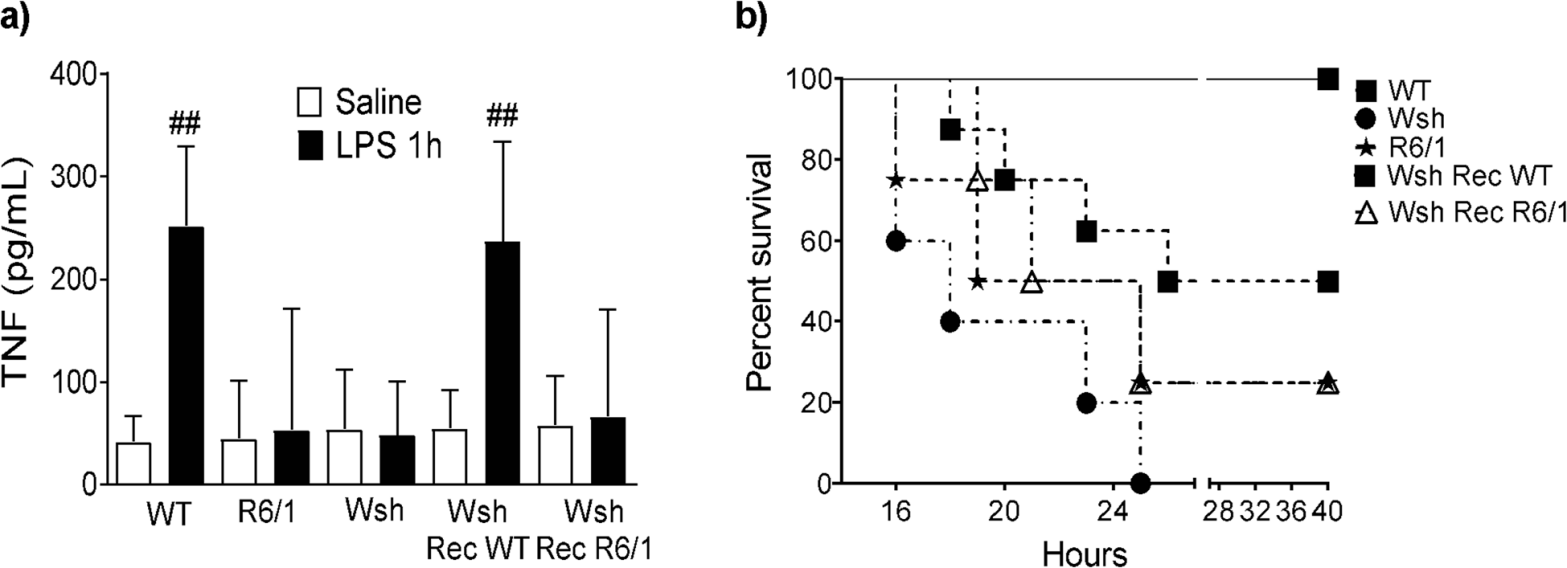
In vivo effects of impaired TLR-4 signaling on R6/1 mast cells. **a** WT, R6/1, Wsh, Wsh reconstituted with WT BMMCs (Wsh Rec WT), and Wsh reconstituted with R6/1 BMMCs (Wsh Rec R6/1) mice were administered i.p. with either saline solution (white bars) or LPS (black bars) (1 mg/kg). One hour later, the mice were euthanized, and peritoneal washes were obtained. TNF was quantified by ELISA in peritoneal washes. Data shown in the graph were obtained from eight WT animals, six R6/1 mice, ten Wsh mice, 12 Wsh Rec WT mice, ten Wsh Rec R6/1 mice per each treatment. Data are presented as the mean ± SD. **b** Same experimental groups (6 animals each) mentioned in (**a**) were i.p. administered with a sub-lethal dose of LPS (80 mg/kg) and the survival was monitored for 40 h. Each point on the lines represent the accumulated death rate. ^##^*p* < 0.01 vs. vehicle-treated animals as determined by two-way ANOVA and post hoc Tukey’s test

To determine whether the impaired TNF secretion in R6/1 BMMCs could be relevant in a mast cell-dependent TLR4-triggerd inflammatory response in vivo, the well-established model of endotoxemia by intraperitoneal administration of LPS in mice [53] was used (Fig. 7a). As previously reported, LPS administration to WT mice induced the release of TNF (252 ± 29.44 pg/mL 1 h after stimulus). In contrast, significantly lower TNF amounts were detected in peritoneal washes from R6/1 mice after LPS stimulus (53.3 ± 118 pg/mL). The participation of MCs in LPS-induced early TNF release was thus confirmed, since TNF was not detected in peritoneal washes from MC-deficient mice (Wsh) but it was restored in Wsh mice reconstituted with WT BMMCs (WshRecWT). However, when Wsh mice were reconstituted with BMMCs derived from R6/1 animals (WshRecR6/1), no TNF secretion was observed after LPS challenge.

To test whether impaired TNF secretion in R6/1 MCs could lead to a reduced protective reaction in sepsis, the model of endotoxemia by i.p. injection of a sub-lethal LPS dose was used [42]. Survival curves were built for WT, R6/1, Wsh, WshRecWT, and WshRecR6/1 mice after LPS administration (80 mg/kg). As shown in Fig. 7b, all WT animals survived 40 h after challenge, whereas 100% of Wsh mice died within 24 h after LPS administration. Wsh mice reconstituted with WT MCs showed an improved survival (about 50%) after 40 h. However, R6/1 mice and Wsh mice reconstituted with R6/1 MCs showed survival rates of 25% 40 h after challenge. These results indicate that, while R6/1 MCs still contributed to the protective immune reaction against LPS administration, they were significantly less efficient than WT MCs.

## Discussion

Aberrant inflammatory reactions in HD have been linked to defects in the function of innate immune cells caused by the expression of mHtt. MCs are key players in inflammatory responses due to their capacity of secreting several pro- and anti-inflammatory mediators [6]. The production of cytokines by this cell type depends on various signaling processes that connect specific stimuli and receptor activation to cytoskeletal rearrangements, calcium mobilization, activation of transcription factors, protein synthesis, and vesicular trafficking [20]. Htt participates in processes associated to signaling like receptor internalization, kinase activation, and gene transcription in different cell types [54]. Herein, we explored the effects of the expression of mHtt on the function of MCs by comparing canonical mast cell-dependent responses in BMMCs derived from WT mice with mHtt-expressing R6/1 mice.

The main findings herein reported are (1) the expression of mHtt does not alter the release of β-hexosaminidase triggered by FcεRI receptor in BMMCs, but it does interfere with the accumulation of IL-10, TGF-β, and TNF mRNAs and with TNF secretion mediated by TLR-4 receptor; (2) the expression of mHtt prevents the TLR4-mediated phosphorylation of IKK and increases the basal phosphorylation levels of the NFκB p65 subunit; (3) mHtt prevents the LPS-induced ERK1/2 phosphorylation, the activation of ELK-1, and the accumulation of *c-fos* mRNA, being those effects accompanied by a decrease in dynamin-dependent TLR-4 receptor vesicular trafficking; and (4) mHtt significantly impairs the protective, MC-dependent innate responses triggered by TLR-4 receptor in vivo*.*

Culturing bone marrow cells from R6/1 mice in the presence of IL-3 and SCF produced about 20% less mature BMMCs than those obtained from WT mice. Our results are in line with those reporting a negative influence of mHtt expression on progenitor cell differentiation, as it has been observed in the division of cortical progenitor cells and the development of the mouse neocortex [55], or in mammary stem cell division and differentiation [55]. Although differences in the initial number of progenitor cells or an altered proliferation capacity of MC precursors in R6/1 bone marrow cannot be ruled out, no differences were apparent between R6/1 mouse-derived mature BMMCs and those obtained from WT mice: they showed the same size and mean number of granules. Additionally, both cell phenotypes expressed FcεRI and TLR-4 receptors and were able to degranulate and secrete β-hexosaminidase in response to IgE/Ag complexes.

LPS-dependent stimulation of BMMCs results in the production of several cytokines, being TNF one of the best characterized since it is rapidly produced after TLR-4 receptor triggering [5, 44, 53]. The molecular events that lead to TNF mRNA production after TLR-4 receptor stimulation have been described in innate immune cell types like macrophages and DCs [56, 57]; transcription factors like AP-1, NFκB, SP1, ELK-1, and NFAT are activated in these cell types [58]. Pre-incubating BMMCs with the IKK inhibitor BAY117085 did not affect LPS-induced TNF mRNA accumulation, but the ERK1/2 inhibitor FR180204 blocked it. Our data are consistent with those indicating that NFκB-binding sites in the TNF gene promoter are not the most relevant ones for gene transcription in MCs. Indeed, an AP-1 transcription factor complex formed by fosB, c-jun, junD, and ATF2 has been shown to bind the TNF promoter in this cell type [59]. Our results coincide with the current knowledge that ERK-dependent transcription factors, but not NFκB, participate in TNF gene transcription in MCs. For example, LPS induces TNF production through the Raf-1/MEK-MEK2/ERK1/2 pathway in monocytes [60]. On the other hand, it has been shown that the − 200 nt proximal TNF gene promoter is capable of driving transcription in response to several stimuli, including LPS, in humans and mice [61]. Four binding sites for ETS/ELK, among other proteins (such as NFAT and Sp1), have been identified in that proximal promoter [61]. Two of the three identified NFκB binding sites are located outside the proximal promoter, and several studies have found that NFκB, while involved in TNF gene regulation, is not important as a transcription factor for TNF upon LPS challenge or other immune stimuli [58].

The expression of mHtt in R6/1 BMMCs was linked to a decreased LPS-dependent IKK phosphorylation, along with increased levels of p65 NFκB activation (as measured by its phosphorylation in the Ser-536 residue). p65 NFκB subunit (RelA) can be phosphorylated by IKKα or IKKβ, and by IKKε or TBK-1 [62]. Our data suggest that a different kinase (other than IKKα/β) could be involved in p65 phosphorylation in BMMCs, and the activation of that kinase seems to be dependent on the presence of mHtt.

Other research groups have studied cultured cells expressing mHtt and striatal cells from HD transgenic mice; in contrast with our findings, those groups reported that soluble mHtt activates IKK, which in turn upregulates the NF-κB signaling pathway [63]. Additionally, decreasing mHtt levels by siRNA in monocytes/macrophages from HD patients was shown to ameliorate NF-κB transcriptional dysregulation and downregulate the expression of pro-inflammatory cytokines [64]. Our data, however, are in agreement with those reporting that the expression of the N171-N-terminal fragment of mHtt (containing 82 glutamine repeats) in cultured microglial cells leads to a decreased production of pro-inflammatory cytokines and nitric oxide generation in response to LPS [61]. Our results strongly suggest that the expression of mHtt has differential effects on NFκB signaling among distinct immune cell lineages. The observed impairment in TLR-4 signaling in the presence of mHtt was extensive to the synthesis of the mRNAs of anti-inflammatory cytokines IL-10 and TGF-β, leading to the hypothesis that by lowering the synthesis of anti-inflammatory cytokines in MCs in response to innate immune stimulus, mHtt expression could contribute to the systemic pro-inflammatory phenotype observed in HD patients. Future research addressing the secretion of negative regulators of inflammation will give light in the complex immune phenotype observed in the disease.

In concordance with the decreased accumulation of TNF mRNA after LPS stimulus, mHtt-expressing BMMCs showed an impaired activation of the TLR4-dependent ERK1/2-ELK1-cFos axis. Remarkably, the activation of that signaling cascade in WT cells required dynamin-dependent TLR-4 receptor vesicular trafficking. Our results are in line with those studies indicating that the activity of dynamin is necessary for the activation of that kinase [65]. For example, dynasore blocks CCL2 and phorbol-myristate acetate-induced ERK1/2 activation in a step located between PKC and that MAPK in HEK293 cells [65], suggesting that intracellular vesicle trafficking is coupled to the formation of the activation complex of MAPK triggered by distinct receptors. Also, dynamin-dependent internalization seemed to be required even for basal ERK1/2 phosphorylation in MCs, because dynasore treatment significantly decreased the phosphorylation rates of that kinase. Since BMMCs used in this study were sensitized with IgE and it has been shown that monomeric IgE slightly increases the basal phosphorylation of ERK1/2 [66], our results indicate that ERK1/2 activation after FcεRI receptor triggering by monomeric IgE could also require intracellular, dynamin-dependent, vesicle trafficking.

Regarding to the defective LPS-induced ERK1/2 activation in mHtt expressing BMMCs, our results are in concordance with previous studies reporting that Htt participates in MAPK activation (specifically on ERK1/2-dependent pathway) [67, 68]. In particular, our data strongly suggest that Htt plays a similar role in receptor internalization and retrograde transport of specific receptors in MCs to that observed in striatal neurons, where Htt participates in the retrograde transport of the TrkB receptor, required for ERK1/2 activation and *c*-*fos* transcription [27].

Our results show that TLR-4 is mainly located in the cytoplasm of BMMCs, and it is translocated to perinuclear zones upon LPS stimulus. Those regions have been proposed to form a “perinuclear cloud” [69] where endosomes are recycled and redistributed to various cell compartments. Our data are in line with those reported by Okumura et al. (2003) showing that, although TLR-4 can be detected in the plasma membrane by flow cytometry, it is mainly located in the cytoplasm of peripheral blood-derived and lung human MCs. Interestingly, TLR-4 receptor was found near the cell nucleus in those cellular preparations [70]. Also, an intracellular location of TLR-4 was observed by confocal microscopy in the human MC line HMC-1 [71], and a low membrane expression of that receptor was found in bone marrow-derived macrophages, being this membrane expression significantly decreased 2 h after LPS treatment [72]. Finding TLR-4 receptor in this location of BMMCs is also consistent with that reported for peripheral blood-derived DCs, where this receptor is not detected on the plasma membrane; instead, it is found in a tubo-vesicular pattern that is more intense in a region near the nucleus, associated with the Golgi apparatus and co-localized with α-tubulin. In DCs, microtubules have been suggested to act as transport tracks for TLR vesicles [73].

Internalization of TLR-4 receptor has been associated to the activation of the TRIF/TRAM-dependent signaling cascade [69, 74] in monocytes and dendritic cells. The current model for TLR-4 signal transduction pathway proposes the activation of the MyD88/TIRAP-dependent MAPK and IKK phosphorylation from the plasma membrane before receptor endocytosis, which is necessary for a second wave of gene transcription depending on IRF3 [72]. Our results indicating that a dynamin-mediated vesicular trafficking or of TLR-4 receptor is needed for ERK1/2 and ELK-1 activation, together with *c*-*fos* mRNA transcription in BMMCs deepens our knowledge on the signal transduction pathways triggered by LPS in these cells, in which a lack of the TRIF/TRAM-mediated TLR-4 signaling axis has been reported [72]. Altogether, our data support the notion that an intracellular location of TLR-4 receptor does not necessarily lead to TRIF-TRAM-dependent events but is required for the MyD88-dependent pathway to function [75].

R6/1 mice and Wsh mice reconstituted with R6/1 BMMCs failed to show the MC-dependent early TNF production after i.p. injection of LPS, showing that the impaired activity in R6/1 MCs are observable in vivo. Since TNF derived from MCs (unlike other cell types) has been associated to a protective response against gram-negative bacterial infections in mice [5, 76, 77], the impaired production of TNF resulted in an increased mortality in R6/1 mice and Wsh mice reconstituted with R6/1 BMMCs after a sub-lethal LPS dose. This result suggests that the expression of mHtt significantly impairs early, MC-dependent, innate immunity responses to TLR-4 receptor ligands in vivo*.*

Recent data supports a significant role of MCs on neuroinflammation, since stabilization of brain MCs diminish LPS-induced microglial activation [78], suggesting that TLR-4 receptor expressed in MCs could be an important element in the regulation inflammatory reactions in the brain. Our data suggest that the expression of mHtt alters MC-driven inflammatory reactions that could be relevant to HD and other neurodegenerative diseases.

## Conclusions

**Fig. 8:**
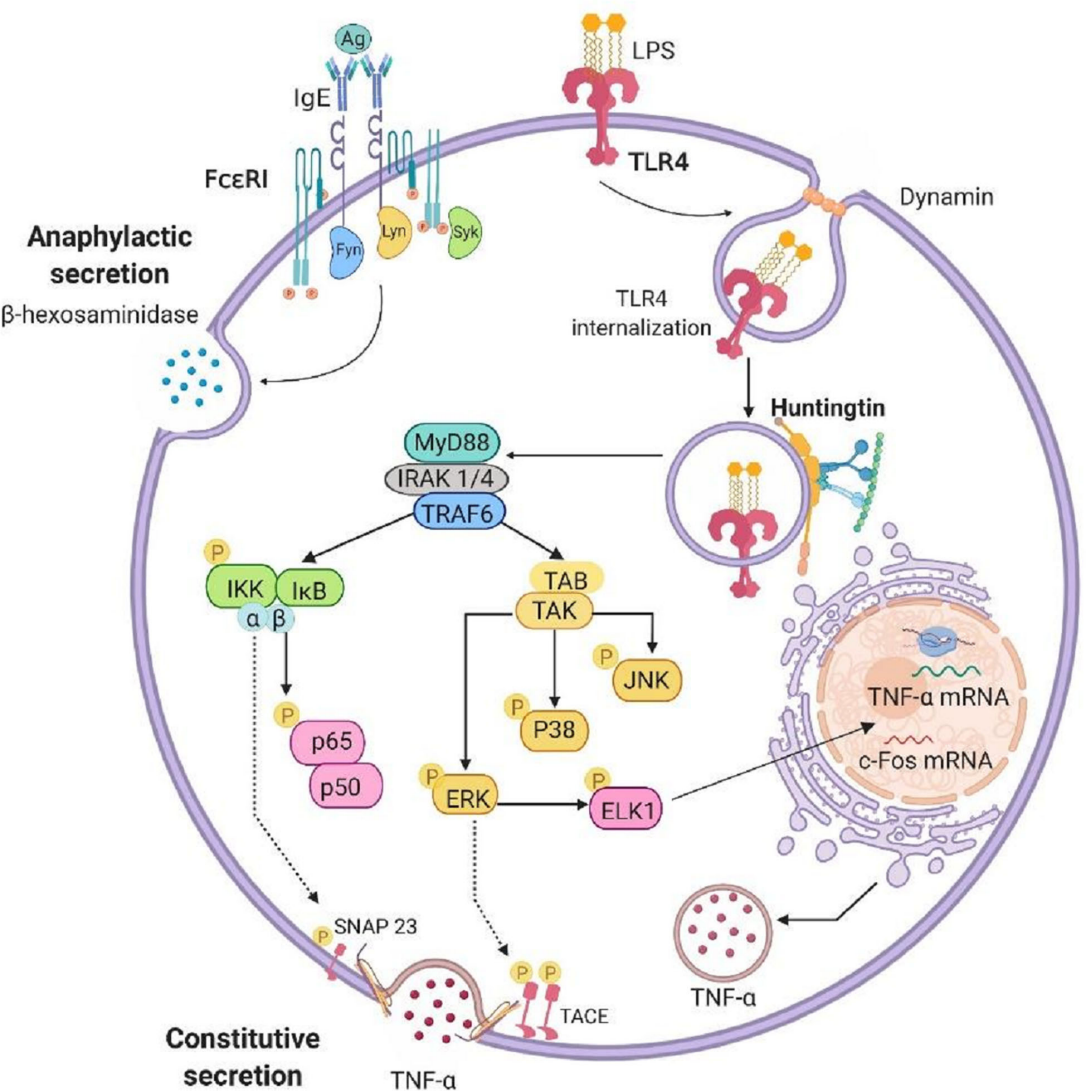
Pleiotropic functions of Htt in the TLR-4 signal transduction system in BMMCs. Upon LPS stimulus, TLR-4 is translocated to the perinuclear area in Htt-containing vesicles by a mechanism depending on the activity of dynamin. This process leads to the activation of the ERK1/2-ELK-1 signaling pathway, which is involved in the accumulation of *c*-*fos* and TNF mRNAs, along with the secretion of TNF via TACE activation. The expression of mHtt in BMMCs not only impairs the ERK1/2 signaling cascade, but also decreases IKK phosphorylation, which has been associated with the phosphorylation of SNAP23 and TNF secretion upon TLR-4 triggering in this cell type. Anaphylactic degranulation triggered by FcεRI seems to be independent on the function of Htt

This work shows, for the first time, that the expression of mHtt alters the internalization of TLR-4 receptor, required for the activation of the ERK1/2-Elk1-c-Fos signaling axis in MCs. Thus, mHtt expression blocks the TLR4-dependent synthesis of pro-and anti-inflammatory cytokines in that cell type (Fig. 8), affecting MC-dependent inflammatory reactions in vivo. Finally, our data suggest a significant impairment in innate immunity-triggered responses of MCs that express mHtt, which could contribute to the altered inflammatory phenotype observed in HD patients.

## Supplementary information

**Additional file 1: FigureS1.** Growth of bone marrow cultures and cell viability of WT and R6/1 BMMCs. a) Total bone marrow from tibias and femurs from WT and R6/1 mice was seeded in a T75 flask with RPMI medium, supplemented with IL-3 and cultured as described in the Methods section. Cells in culture supernatants were counted. Data are presented as the mean ± SD from at least 20 cultures obtained from the same number of mice. b) One million BMMCs of each genotype were re-suspended in 1 mL of supplemented RPMI, mixed with the Muse™ Count Viability Kit reagent (Millipore) and analyzed for viability with the Muse™ Cell Analyzer. ***p < 0.001 vs. WT values; &&&*p* < 0.001 vs. the value at time zero or in non-stimulated cells as determined by two-way ANOVA and post hoc Tukey’s test.

**Additional file 2: Figure S2.** Effect of inhibition of IKK or ERK1/2 on LPS-induced TNF mRNA synthesis. WT BMMCs were pre-incubated for 15 min at 37 °C with a) BAY117085 (10 μM) or b) FR180204 (10 μM) and then stimulated with LPS (500 ng/mL). The cells were collected at different times post-stimulus and total RNA was extracted to detect TNF mRNA by RT-PCR. The upper part of each panel shows a representative image from at least three independent experiments, while TNF mRNA accumulation is shown in the lower panel. Data are presented as the mean ± SD from at least three independent experiments performed with different cell cultures. &*p* < 0.05, && *p* < 0.01, vs. the value at time zero or in non-stimulated cells; #*p* < 0.05 vs. vehicle-treated cells as determined by two-way ANOVA followed by Tukey’s test.

**Additional file 3: Figure S3.** mHtt expression prevents TLR4-triggered IL-10 and TGF-β mRNA synthesis. a) Time-course of IL-10 mRNA expression in WT and R6/1 cells in response to LPS. b) Time-course of TGF-β mRNA expression in WT and R6/1 cells in response to LPS. Upper panels show an image obtained from a representative experiment, and lower panels show densitometric analysis of different experiments. Data are presented as the mean ± SD from at least three experiments performed with independent cultures. *p < 0.05, **p < 0.01, ***p < 0.001 vs. WT values; &*p* < 0.05, &&*p* < 0.01, &&&*p* < 0.001 vs. the value at time zero or in non-stimulated cells as determined by two-way ANOVA followed by Tukey’s test.

## Data Availability

All datasets generated and/or analyzed in this study are available from the corresponding author on reasonable request.

## Abbreviations

BMMC: Bone marrow-derived mast cells
DAMPs: Damage-associated molecular patterns
DCs: Dendritic cells
ELK1: ETS transcription factor
ERK: Extracellular-signal-regulated kinase
FcεRI: High-affinity IgE receptor
HD: Huntington’s disease
Htt: Huntingtin
IKK: IκB kinase
IL-10: Interleukin 10
IL-3: Interleukin 3
IL-6: Interleukin 6
LPS: Lipopolysaccharide
mHtt: Mutated Huntingtin
NFκB: Nuclear factor kappa-light-chain-enhancer of activated B cells
PAMPs: Pathogen-associated molecular patterns
TGFβ: Transforming growth factor beta
TLR4: Toll-like receptor 4
TNF: Tumor necrosis factor
Wsh: MC-deficient *c-Kit*^*Wsh/Wsh*^
