# Supplementary figures and images for: Mutant Huntingtin affects toll-like receptor 4 intracellular trafficking and cytokine production in mast cells
Source: J Neuroinflammation. 2020 Mar 27;17:95. doi: 10.1186/s12974-020-01758-9 (PMC7102443; doi:10.1186/s12974-020-01758-9)

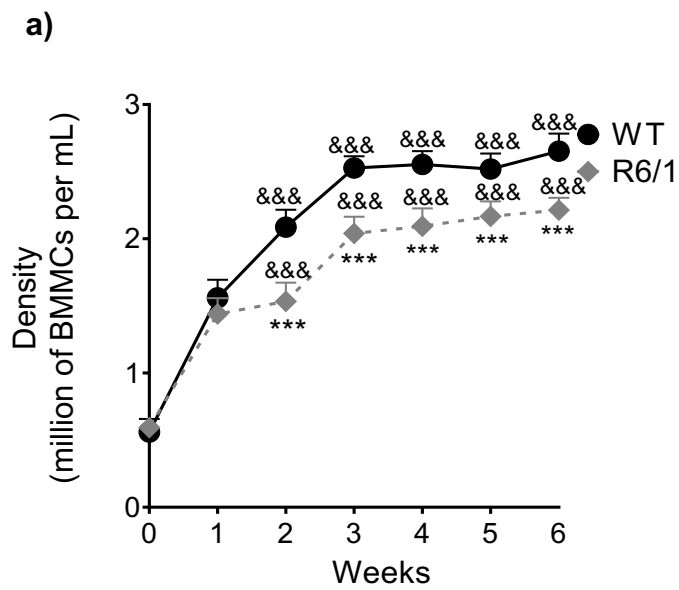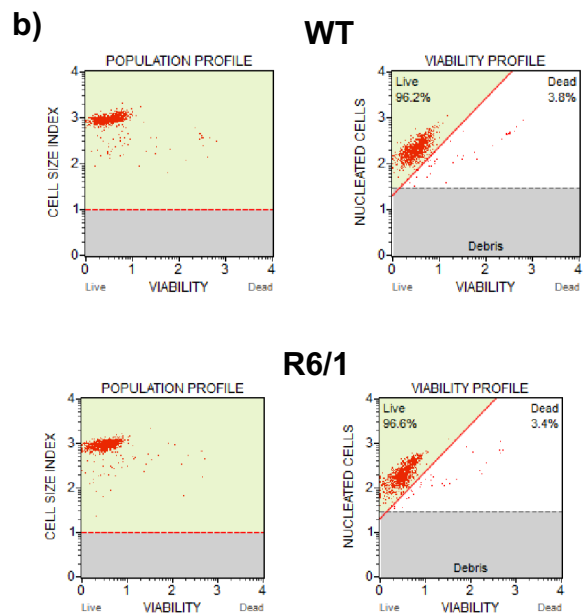

Supplement: Supplementary file 1 — Additional file 1: FigureS1. Growth of bone marrow cultures and cell viability of WT and R6/1 BMMCs. a) Total bone marrow from tibias and femurs from WT and R6/1 mice was seeded in a T75 flask with RPMI medium, supplemented with IL-3 and cultured as described in the Methods section. Cells in culture supernatants were counted. Data are presented as the mean ± SD from at least 20 cultures obtained from the same number of mice. b) One million BMMCs of each genotype were re-suspended in 1 mL of supplemented RPMI, mixed with the Muse™ Count Viability Kit reagent (Millipore) and analyzed for viability with the Muse™ Cell Analyzer. ***p < 0.001 vs. WT values; &&&p < 0.001 vs. the value at time zero or in non-stimulated cells as determined by two-way ANOVA and post hoc Tukey’s test. [file 12974_2020_1758_MOESM1_ESM.pdf]

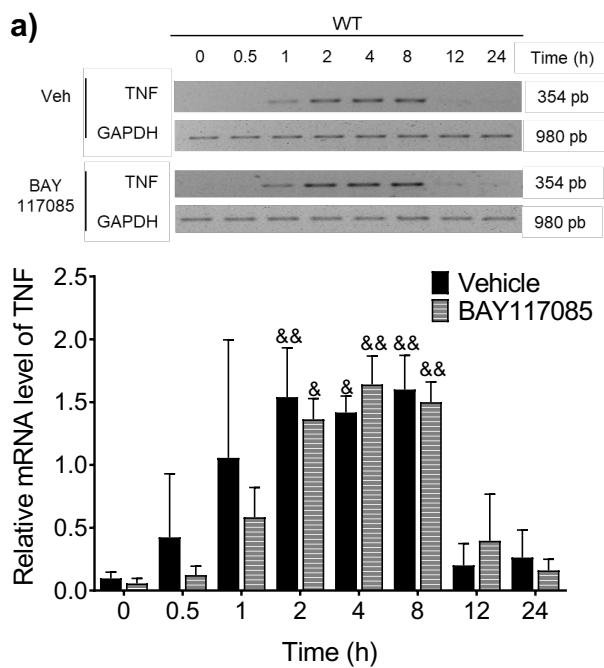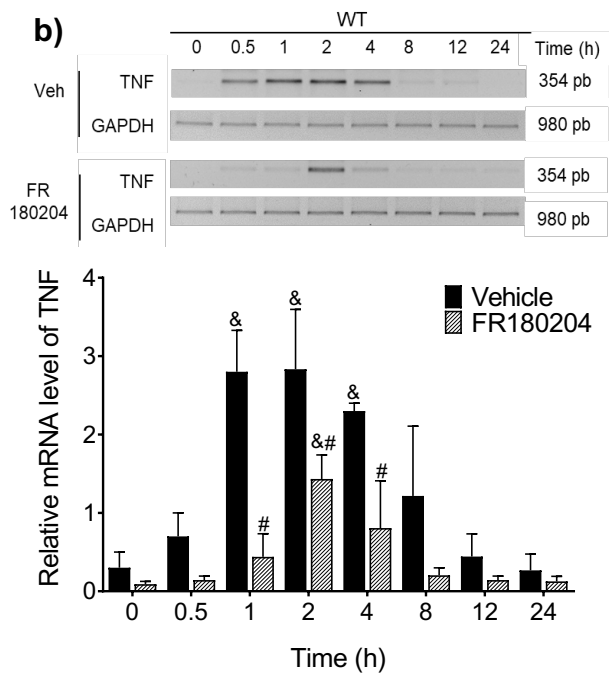

Supplement: Supplementary file 2 — Additional file 2: Figure S2. Effect of inhibition of IKK or ERK1/2 on LPS-induced TNF mRNA synthesis. WT BMMCs were pre-incubated for 15 min at 37 °C with a) BAY117085 (10 μM) or b) FR180204 (10 μM) and then stimulated with LPS (500 ng/mL). The cells were collected at different times post-stimulus and total RNA was extracted to detect TNF mRNA by RT-PCR. The upper part of each panel shows a representative image from at least three independent experiments, while TNF mRNA accumulation is shown in the lower panel. Data are presented as the mean ± SD from at least three independent experiments performed with different cell cultures. &p < 0.05, && p < 0.01, vs. the value at time zero or in non-stimulated cells; #p < 0.05 vs. vehicle-treated cells as determined by two-way ANOVA followed by Tukey’s test. [file 12974_2020_1758_MOESM2_ESM.pdf]

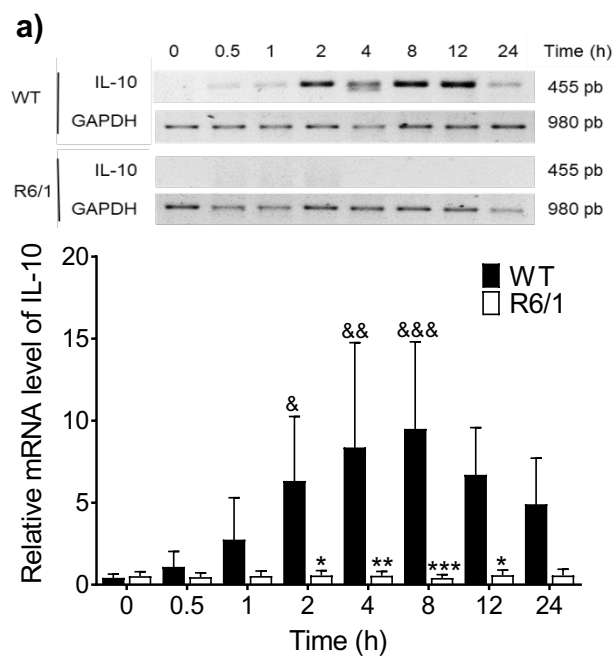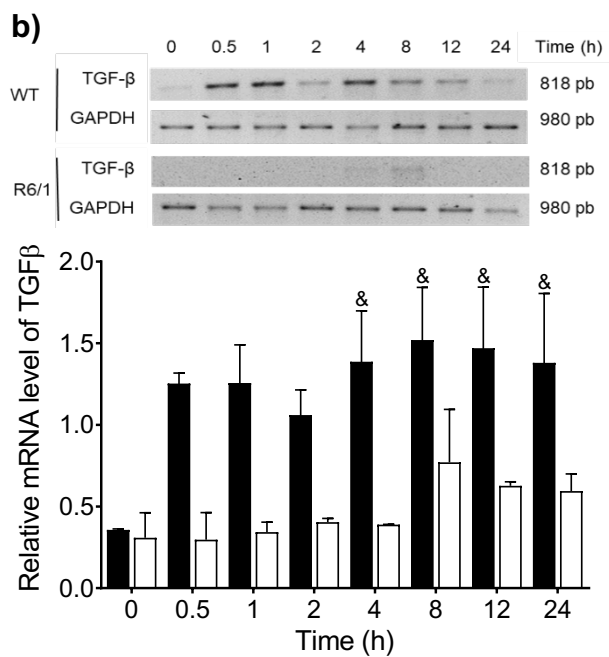

Supplement: Supplementary file 3 — Additional file 3: Figure S3. mHtt expression prevents TLR4-triggered IL-10 and TGF-β mRNA synthesis. a) Time-course of IL-10 mRNA expression in WT and R6/1 cells in response to LPS. b) Time-course of TGF-β mRNA expression in WT and R6/1 cells in response to LPS. Upper panels show an image obtained from a representative experiment, and lower panels show densitometric analysis of different experiments. Data are presented as the mean ± SD from at least three experiments performed with independent cultures. *p < 0.05, **p < 0.01, ***p < 0.001 vs. WT values; &p < 0.05, &&p < 0.01, &&&p < 0.001 vs. the value at time zero or in non-stimulated cells as determined by two-way ANOVA followed by Tukey’s test. [file 12974_2020_1758_MOESM3_ESM.pdf]
